# Cesium activates the neurotransmitter receptor for glycine

**DOI:** 10.3389/fnmol.2023.1018530

**Published:** 2023-05-22

**Authors:** Steffen Fricke, Magnus Harnau, Florian Hetsch, Haoran Liu, Julia Leonhard, Anna Eylmann, Pina Knauff, Han Sun, Marcus Semtner, Jochen C. Meier

**Affiliations:** ^1^Division Cell Physiology, Zoological Institute, Technische Universität Braunschweig, Braunschweig, Germany; ^2^Institute of Pathophysiology, University Medical Center of the Johannes Gutenberg University Mainz, Mainz, Germany; ^3^Structural Chemistry and Computational Biophysics, Leibniz-Forschungsinstitut für Molekulare Pharmakologie, Berlin, Germany; ^4^Institute of Chemistry, Technical University of Berlin, Berlin, Germany; ^5^Psychoneuroimmunology, Max Delbrück Center for Molecular Medicine, Berlin, Germany

**Keywords:** glycine receptor (GlyR), alkali metal, molecular modeling, agonist, electrophysiology

## Abstract

The monovalent cations sodium and potassium are crucial for the proper functioning of excitable cells, but, in addition, other monovalent alkali metal ions such as cesium and lithium can also affect neuronal physiology. For instance, there have been recent reports of adverse effects resulting from self-administered high concentrations of cesium in disease conditions, prompting the Food and Drug Administration (FDA) to issue an alert concerning cesium chloride. As we recently found that the monovalent cation NH_4_^+^ activates glycine receptors (GlyRs), we investigated the effects of alkali metal ions on the function of the GlyR, which belongs to one of the most widely distributed neurotransmitter receptors in the peripheral and central nervous systems. Whole-cell voltage clamp electrophysiology was performed with HEK293T cells transiently expressing different splice and RNA-edited variants of GlyR α2 and α3 homopentameric channels. By examining the influence of various milli- and sub-millimolar concentrations of lithium, sodium, potassium, and cesium on these GlyRs in comparison to its natural ligand glycine (0.1 mM), we could show that cesium activates GlyRs in a concentration- and post-transcriptional-dependent way. Additionally, we conducted atomistic molecular dynamic simulations on GlyR α3 embedded in a membrane bilayer with potassium and cesium, respectively. The simulations revealed slightly different GlyR-ion binding profiles for potassium and cesium, identifying interactions near the glycine binding pocket (potassium and cesium) and close to the RNA-edited site (cesium) in the extracellular GlyR domain. Together, these findings show that cesium acts as an agonist of GlyRs.

## Introduction

Each cell in our body is filled with fluid containing alkali metal salts. These also make up the extracellular fluid, which is considerably different in composition from the intracellular fluid. Sodium and potassium cations are key components in these solutions, enabling the functional vitality of living cells. In neuronal cells, their asymmetrical distribution in the intra- and extracellular spaces is essential for maintaining the resting membrane potential and, thereby, acts as a driving force for the generation of action potentials through opening of their respective ion channels. Imbalances in ion homeostasis on either side of the cell membrane can lead to severe and life-threatening conditions.

While high concentrations of sodium and potassium are commonly found in and around living cells, the occurrence of cesium and lithium is scarce. However, particularly cesium’s physico-chemical properties are comparable to those of potassium, making it an interesting tool in electrophysiological research. In this regard, extracellular cesium is traditionally used as a blocker of voltage-gated potassium channels and as a substitute for potassium ions in intracellular solutions (Clay and Shlesinger, 1984). Extracellular application of cesium ions has also been shown to inhibit the hyperpolarization-activated current (I_*h*_), making it a popular blocker of this specific type of ion channel (Thoby-Brisson et al., 2000; Rateau and Ropert, 2006). The mechanism behind this blockage seems to involve voltage- and concentration-dependent channel blockade (DiFrancesco, 1982). Additionally, monovalent cations, such as potassium, rubidium and cesium exhibit distinct voltage-dependent gating behavior in various members of the two-pore domain potassium channels (Schewe et al., 2016). Most importantly, cesium was found to activate the same chloride channel as glycine, which sparked discussions about their association already three decades ago (Lewis et al., 1989; Smith and McBurney, 1989). Cesium chloride is advertised as an alternative therapy agent for different types of cancer, but prolonged self-administration leads to severe health decline involving neuronal and cardiovascular dysfunction (Dalal et al., 2004; Sessions et al., 2013). Recently, the FDA published alerts with regard to cesium (U.S. Food and Drug Administration, 2018, 2020).

The neurotransmitter receptor for glycine (GlyR) is a chloride-permeable ion channel belonging to the family of ligand-gated pentameric Cys-loop receptors. Homopentamers are composed of subunits α1–4, whereas the heteropentamer includes three β subunits (Legendre, 2001; Betz and Laube, 2006). The latter has mostly structural functions via binding of gephyrin (Meyer et al., 1995; Meier et al., 2001). The α1 subunit contributes to functions in the spinal cord and brainstem, but it is not expressed in the hippocampus (Legendre, 2001; McCracken et al., 2017). The subunit α4 is a pseudogene that is most likely non-functional in humans (Leacock et al., 2018). Therefore, we focused on the GlyRs α2 and α3, which are present in forebrain structures such as the hippocampus (McCracken et al., 2017).

The molecular and functional diversity of GlyRs is further extended by alternative splicing and C-to-U RNA editing, resulting in proline-to-leucine substitution (Meier et al., 2005). The GlyR splice variants α2A and α2B have two different amino acids in the external N-terminal domain, which affects agonist efficacy (Miller et al., 2004). GlyR α3 splice variants differ by a short amino acid sequence in the intracellular loop between transmembrane domains 3 and 4, which is encoded by exon 8A (Nikolic et al., 1998). This has an impact on their location in the cell because the long splice variant α3L interacts with the Sec8 trafficking protein, leading to trafficking of GlyR α3L clusters to presynaptic sites (Winkelmann et al., 2014). At the presynapse, GlyRs were found to increase neurotransmitter release and thereby the impact of individual neurons, impairing neuronal network homeostasis (Meier et al., 2014; Winkelmann et al., 2014). The short GlyR α3K variant is diffusely distributed in the neuronal plasma membrane (Eichler et al., 2009; Notelaers et al., 2012, 2014a,b; Lemmens et al., 2022) and mediates tonic inhibition of neuronal excitability (Eichler et al., 2008).

C-to-U RNA editing of GlyR-coding mRNAs was shown to be increased in patients with intractable temporal lobe epilepsy (TLE) (Eichler et al., 2008), as it generates receptor gain-of-function protein variants (Meier et al., 2005; Legendre et al., 2009). Neuronal gain of function through presynaptic function of RNA-edited GlyR α3L leads to cognitive dysfunction associated with explicit memory deficits and reduced extinction of contextual fear memory, when expressed in excitatory neurons or parvalbumin-positive interneurons, respectively (Winkelmann et al., 2014; Çaliskan et al., 2016), while tonic inhibition through RNA-edited GlyR α3K causes neurodegeneration (Eichler et al., 2008; Winkelmann et al., 2015). GlyR α3 is furthermore involved in inflammatory pain sensitization (Harvey et al., 2004).

In this study, we conducted systematic whole-cell voltage clamp electrophysiology using different alkali metal ions with HEK293T cells that expressed various splice and editing variants of GlyR α2 and α3 homopentamers. We show that GlyRs, which have undergone post-transcriptional C-to-U RNA-editing, are more sensitive to the alkali metal ion cesium (Cs^+^) in an agonistic manner than unedited GlyRs. In contrast, other alkali metal ions, such as Li^+^, Na^+^ and K^+^ do not evoke GlyR current responses. Using atomistic molecular dynamic (MD) simulations, we identified two major binding sites for K^+^ and Cs^+^ in the extracellular domain of the open-state of GlyRs, which are located near the glycine binding pocket and the RNA-edited site. These results shed light on possible activation mechanisms of GlyR by Cs^+^.

Altogether, the findings of this and earlier studies (Lewis et al., 1989; Smith and McBurney, 1989) reveal that Cs^+^ is an agonist of GlyRs, which can help to provide scientific support for the FDA alert and may contribute to a better understanding of the adverse health effects associated with the intake of Cs^+^, although these seem to result primarily from cardiovascular dysfunction.

## Materials and methods

### Culture and transfection of HEK293T cells

HEK293T cells (DSMZ, ACC 635) were cultured in T25 culture flasks filled with 5 ml DMEM^+^ (DMEM, #41965-062, Gibco™) supplemented with 10% fetal calf serum (FCS, #1050064, Life Technologies) and 1% penicillin/streptomycin (#15140122, Life Technologies) at 37°C and 5% CO_2_ in a humidified incubator. Cells were passaged every 2–3 days when 80–90% confluence was reached. HEK293T cells were seeded onto 35 mm culture dishes containing 1.5 ml DMEM^+^ to reach 80–90% confluence for the subsequent transfection with FuGENE^®^ HD Transfection Reagent (Promega, #E2311). Per transfection, 1 μg of DNA was used according to the manufacturer’s protocol. Co-transfection of GlyR-coding plasmids with EGFP-coding plasmid were performed at a ratio of 10:1. However, for some constructs IRES-dependent co-expression of EGFP was used (see Table 1). Cells were incubated with the transfection mix overnight and seeded on 13 mm diameter glass coverslips coated with 0.1% poly-DL-ornithine for 60 min the following day. After incubation at 37°C and 5% CO_2_ for 1–2 h, the cells were sufficiently attached to start patch clamp recordings.

**TABLE 1:** Key resource table.

Reagent or resource	Source	Identifier
**Antibodies**		
**Bacterial and virus strains**		
**Biological samples**		
**Chemicals, peptides, and recombinant proteins**		
Dulbecco’s modified eagle medium (DMEM)	Gibco™	Cat.# 41965039
Poly-DL-ornithine hydrobromide	Sigma	Cat.# P8638100MG
Penicillin/streptomycin	Gibco™	Cat.# 15140122
Heat inactivated fetal bovine serum	Gibco™	Cat.# 10500064
Lithium chloride	Carl Roth	Cat.# 3739.1 batch 2015: 031166098 batch 2021: 201302565 (tested with NMR, Supplementary Figure 2)
Lithium hydroxide	Sigma	Cat.# 254274-10G
Cesium chloride	Carl Roth	Cat.# 8627.1 batch 2015: 494209893 batch 2017: 137256892 batch 2018: 497251204 (tested with NMR, Supplementary Figure 2)
Cesium hydroxide	Fluka	Cat.# 21000-10G-F (tested with NMR, Supplementary Figure 2)
N-Methyl-D-glucamine	Sigma	Cat.# M2004-500g
HEPES	Carl Roth	Cat.# 9105.4
EGTA	AppliChem	Cat.# 67-42-5
D-glucose monohydrate	Sigma	Cat.# G8270-1kg
Calcium chloride dihydrate	Carl Roth	Cat.# T885.2
Potassium chloride	VWR Normapur	Cat.# 26764.260
Sodium chloride	Carl Roth	Cat.# P029.2
Hydrochloric acid 37%	VWR Normapur	Cat.# 20252.290
Sodium hydroxide	VWR Normapur	Cat.# 28244.262
**Critical commercial assays**		
FugeneHD	Promega	Cat.# E2311
**Experimental models: cell lines**		
HEK293T	DSMZ	ACC 635
**Experimental models: organisms/strains**		
**Oligonucleotides**		
**Recombinant DNA**		
GlyR α3K-185P (C-terminal IRES-dependent EGFP-expression)	Corresponding to NCBI NM_001368774.2	Lab ID: 51.41.27
GlyR α3K-185L (C-terminal IRES-dependent EGFP-expression)	Corresponding to NCBI NM_001368774.2	Lab ID: 51.41.9 or 64.97.2
GlyR α3K-185P (no HA-tag)	Corresponding to NCBI NM_001368774.2	Lab ID: 64.35.1
GlyR α3K-185L (no HA-tag)	Corresponding to NCBI NM_001368774.2	Lab ID: 14.104.11
GlyR α3L-185P (C-terminal IRES-dependent EGFP-expression)	Corresponding to NCBI NM_080438.4	Lab ID: 19.15.4 or 55.1.1
GlyR α3L-185L (C-terminal IRES-dependent EGFP-expression)	Corresponding to NCBI NM_080438.4	Lab ID: 19.15.3 or 55.1.2
pEGFP-N1	Clontech, NCBI U55762.1	Lab ID: 16.143.5 or 63.15.3
GlyR α2A-192P	Corresponding to NCBI NM_012568.4	Lab ID: 6.574.1M
GlyR α2A-192L	Corresponding to NCBI NM_012568.4	Lab ID: 14.104.10 or 6.594.2
GlyR α2B-192P	Corresponding to NCBI XP_008771374.1	Lab ID: 14.104.1 or 6.594.4
GlyR α2B-192L	Corresponding to NCBI XP_008771374.1	Lab ID: 14.104.5 or 6.594.3
**Software and algorithms**		
Prism 8	GraphPad	https://www.graphpad.com/
Patchmaster 2 × 90	HEKA Electronik GmbH	https://www.heka.com/downloads/downloads_main.html
IgorPro 6.3.7.2	WaveMetrics Inc.	www.wavemetrics.com
Fiji	Schindelin et al., 2012	https://fiji.sc/#download
Metamorph Imaging Software	Molecular Devices	https://de.moleculardevices.com/products/cellular-imaging-systems/acquisition-and-analysis-software/metamorph-microscopy
Origin8.1G	OriginLab	https://www.originlab.com/
**Other**		
Kwik-Fil borosilicate glass capillaries	World Precision Instruments	Cat.#1B150F-4
13 mm diameter glass coverslips	Hecht Assistent	Cat.#41001113
8 channel perfusion pencil	AutoMate Scientific, Inc.	Cat.#04-08-360

Expression plasmids encoding GlyR α2 and α3 open reading frames were described recently (Förstera et al., 2014; Raltschev et al., 2016) and correspond to *Glra2* (NCBI accession numbers, α2A: NM_012568.4, α2B: XP_008771374.1) and *Glra3* (NCBI accession numbers, α3K: NM_001368774.2, α3L: NM_080438.4). Transcription of GlyR open reading frames is driven by the cytomegalovirus promotor (CMV). GlyR constructs are epitope-tagged (c-myc, GlyR α2; HA, GlyR α3), however, recent work demonstrates that epitope tags inserted at positions corresponding +2 in signal peptide processed mature GlyR proteins do not affect the electrophysiological properties of GlyR, as shown recently even for high molecular weight tags such as different fluorescent proteins (Lemmens et al., 2022). In fact, GlyR α3K channels without epitope tag responded comparably well to cesium as did GlyR α3K channels with an HA tag (Supplementary Figure 1). RNA-edited GlyR variants encode a leucine instead of a proline at position +192 and +185 of the respective signal peptide processed mature GlyR splice variants α2A/B and α3K/L, respectively.

### Patch clamp electrophysiology

Whole-cell voltage clamp was performed using an EPC 7 or an EPC 10 amplifier combined with Patchmaster software (HEKA) under a Nikon Ts2R microscope or Zeiss Axiovert 10. A pE-4000 illumination system (CoolLED) or mercury short-arc lamp (Osram, HBO100) was used to visualize EGFP fluorescence. Patch pipettes were pulled from borosilicate glass capillaries and exhibited resistances from 3 to 7 MΩ when filled with intracellular solution containing (in mM): CsCl (130), NaCl (5), CaCl_2_ (0.5), MgCl_2_ (1), EGTA (5) and HEPES (30); pH 7.2 (CsOH) with an osmolarity of 295 mOsm. Standard extracellular solution (ES) contained (in mM): NaCl (140), KCl (5), MgCl_2_ (1), CaCl_2_ (2), HEPES (10) and glucose (10); pH 7.4 (NaOH) with an osmolarity of 305 mOsm. The N-methyl-D-glucamine (NMDG^+^) extracellular solution contained (in mM): NMDG^+^ (150), MgCl_2_ (1), CaCl_2_ (2), HEPES (30) and glucose (10); pH 7.4 (HCl) with an osmolarity of 316 mOsm. The use of NMDG^+^ was necessary to ensure that no monovalent cations were present in the extracellular solution. Extracellular solutions containing proportional Cs^+^ had the following osmolarities (in mOsm): 0.001 mM Cs^+^ (315), 0.5 mM Cs^+^ (320), 5 mM Cs^+^ (322), 50 mM Cs^+^ (325), and 150 mM Cs^+^ (333). Osmolarity was measured using a semi-micro osmometer (K-7400, Knauer). Addition of MgCl_2_ and CaCl_2_ was necessary to achieve a stable gigaseal formation. IV curves were generated from voltage ramps ranging from -150 to +150 mV applied every 5–10 s (Raltschev et al., 2016). For the analysis of the current–voltage relationships, IV curves recorded immediately before glycine application were extracted and subtracted from the respective IV curves in the presence of glycine or Cs^+^ at the peak of the current amplitude using IGOR Pro software (Raltschev et al., 2016). The peak currents were used to calculate ratios to glycine-mediated current responses (0.1 mM). The changes in liquid junction potential due to the composition of the different solutions were not adjusted online during the experiment. However, the IV curves (shown in Figures 2E, F) were corrected offline for the liquid junction potential of the respective solutions (0.1 mM glycine: 10.529 mV; 0.5 mM Cs^+^: 10.485 mV; 5 mM Cs^+^: 10.098 mV; 50 mM Cs^+^: 6.614 mV; 150 mM Cs^+^: 0.618 mV).

Cells investigated in voltage clamp mode were clamped to a holding potential of −50 mV. Their series resistances (R_s_) were monitored by −5 mV voltage pulses (50 ms) applied every 5 s and lay between 10 and 35 MΩ. R_s_ was not compensated during the experiments. Data were recorded at 20 kHz sampling rate after filtering at 2.8 kHz using a Bessel filter. The experiments were performed at room temperature (24°C). Electrophysiological data were analyzed offline using IGOR Pro software with a custom written tool by M. Semtner (as described in Raltschev et al., 2016).

Cells expressing GlyR variants were identified by their EGFP fluorescence. Extracellular solutions were applied gravity-driven using a perfusion pencil with a 360 μm tip (AutoMate Scientific, #04-08-250) to obtain rapid fluid exchange rates (<1 s). For the analysis of alkali metal ion-elicited currents, NMDG^+^ extracellular solution was supplemented proportionally with cesium, lithium, potassium or sodium chloride at the various concentrations tested. Cells were opened in the presence of ES followed by application of NMDG^+^ solution. If various concentrations of alkali metal ions were utilized in an experiment, NMDG^+^ solution without alkali metals was applied in between the respective alkali metal ion concentrations.

### NMR spectroscopy

To check whether LiCl, CsCl, or CsOH solutions were contaminated with glycine, NMR experiments were conducted using a Bruker AV-III 600 MHz spectrometer equipped with a 5 mm room temperature QXI probe head (H,C,N,P) with z-Gradient. Standard Bruker pulse sequences were used for conducting 1D ^1^H experiments at 298 K, with 2000 scans performed for each sample. The spectra were recorded with a spectral width of 16.6 ppm. Chemical shifts (δ) were referenced using the MeOH-*d*_4_ signal. Glycine was used as a reference for the NMR measurements and was purchased from Sigma Aldrich with a purity of =99% (Supplementary Figure 2).

### Atomistic molecular dynamics simulation

The homology structure of GlyR α3 was generated using SWISS-MODEL (Waterhouse et al., 2018) based on the amino acid sequence (AAK51962) and the Cryo-EM structure of the open conformation of zebrafish GlyR α1 homo-pentamer (PDB ID: 6UD3; Kumar et al., 2020). To prepare the molecular dynamic simulation setup, we embedded the GlyR α3 into a POPC lipid membrane using CHARMM-GUI (Jo et al., 2008). All endogenous ligands, such as glycine, were removed before the simulations. All titratable residues of the protein were protonated according to their standard protonation state at pH 7. We prepared two simulation setups, one with 150 mM KCl and the other with CsCl, respectively. The simulations were performed using three different force field and water model combinations: (i) Charmm36m (Huang et al., 2016) + TIP3P (Jorgensen et al., 1983); (ii) Amber19SB (Tian et al., 2020) + TIP3P; (iii) Amber19SB + OPC (Izadi et al., 2014). The system was equilibrated in six steps using default scripts provided by the CHARMM-GUI webserver (Lee et al., 2016). A time step of 2 fs was used for the 1.875 ns equilibration. For each simulation setup with K^+^ and Cs^+^, respectively, we conducted three independent runs of production simulations, each for 300 ns using an integration time step of 2 fs. Short-range electrostatic interactions were calculated with a cutoff of 1.0 nm, and long-range electrostatic interactions were treated using the particle mesh Ewald method (Darden et al., 1993). The cutoff for van der Waals interaction was set to 1.0 nm. The simulations were performed at 300 K with an enhanced Berendsen thermostat (GROMACS V-rescale thermostat, Bussi et al., 2007). The Parrinello-Rahman barostat (Parrinello and Rahman, 1981) was employed to maintain the pressure within the system remaining at 1 bar. All bonds were constrained with the Linear Constraint Solver (LINCS) algorithm (Hess et al., 1997).

### Statistical analysis

Statistical analysis of current amplitudes was performed using Origin 8.1G and Prism 8 software. Data were checked for a normal distribution and, if a normal distribution was assumed, analyzed by One-way ANOVA, Repeated-measures ANOVA with respective post-tests or if normality was rejected, analyzed by Mann–Whitney test. Non-linear regression of the concentration-response curves was performed in Prism 8 using the Hill slope. As GlyR responses to Cs^+^ were not always saturating, the determined Hill coefficients should be considered with care, and no EC_50_ values were extracted for that reason.

## Results

### Cesium activates α3L-GlyRs

**FIGURE 1:**
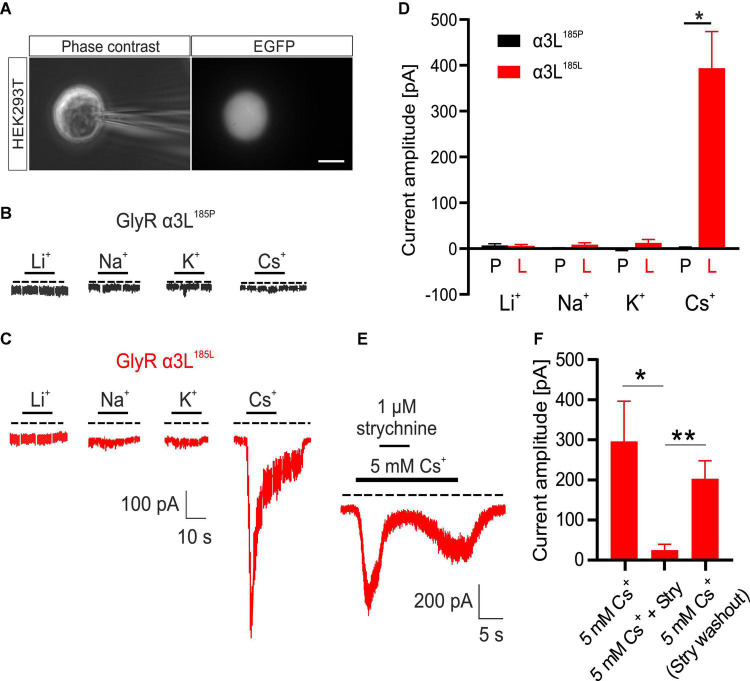
Effect of various alkali metals on RNA-edited and -unedited GlyR α3L. **(A)** Example images of a patch clamped HEK293T cell transfected with GlyR α3L^185L^ under phase-contrast illumination (left panel) and excitation at 490 nm to detect EGFP (right panel). Scale bar is 10 μm. **(B,C)** Representative patch clamp recordings from HEK293T cells expressing GlyR α3L^185P^ [**(B)**, black] or the RNA-edited GlyR α3L^185L^ [**(C)**, red] at a holding potential of −50 mV. 5 mM Li^+^, Na^+^, K^+^ or Cs^+^ were applied as indicated. The dashed lines indicate 0 pA. The annotated bars above the traces indicate application of the tested alkali ions. Scale bar is 10 s horizontal, 100 pA vertical. Note that responses to –5 mV voltage pulses (50 ms) applied every 5 s were cut out of the traces. **(D)** Quantification of the maximum current amplitude of HEK293T cells expressing GlyR α3L^185P^ (black) or GlyR α3L^185L^ (red) during the application of 5 mM Li^+^ (α3L^185P^, *n* = 10; α3L^185L^, *n* = 14), Na^+^ (α3L^185P^, *n* = 6; α3L^185L^, *n* = 7), K^+^ (α3L^185P^, *n* = 6; α3L^185L^, *n* = 7), or Cs^+^ (α3L^185P^, *n* = 5; α3L^185L^, *n* = 15). **(E)** Representative recording of a HEK293T cell expressing GlyR α3L^185L^ treated for 20 s with 5 mM Cs^+^. After the initial current response, 1 μM strychnine was co-applied for 5 s. Note that upon strychnine washout, the current response recovered due to the presence of 5 mM Cs^+^. The dashed line indicates 0 pA. Scale bar is 10 s horizontal, 100 pA vertical. **(F)** Quantification of the maximum current amplitude of HEK293T cells expressing GlyR α3L^185L^ in the presence of 5 mM Cs^+^ and 1 μM strychnine. Currents were measured right before strychnine application, at the plateau phase of strychnine effect as well as right after washout of strychnine (α3L^185L^, *n* = 8). Data are presented as mean ± SEM. *p < 0.05, **p < 0.01.

We have previously shown that NH_4_^+^ at low millimolar concentrations activates RNA-edited GlyRs expressed either heterologous in HEK293T cells or intrinsically in primary neuronal cultures (Kankowski et al., 2018). As alkali metal ions are similar to NH_4_^+^ with respect to diameter and charge, we tested their effect on currents through GlyR α3L. To this end, we overexpressed either GlyR α3L^185P^ or RNA-edited GlyR α3L^185L^ in HEK293T cells and performed whole-cell voltage clamp recordings at a holding potential of −50 mV (Figures 1A–C). The standard extracellular solution in these experiments contained the monovalent cation NMDG^+^ at a concentration of 150 mM. In order to test different alkali metals for their ability to activate GlyR α3L, we applied solutions proportionally substituted with 5 mM of either Li^+^, Na^+^, K^+^ or Cs^+^ for NMDG^+^ (Figures 1B–D). GlyR α3L^185*L*^-expressing HEK293T cells responded to 5 mM Cs^+^ (0.394 ± 0.080 nA, *n* = 15) whereas GlyR α3L^185P^ did not (0.002 ± 0.001 nA, *n* = 5, *p = 0.013). Li^+^, Na^+^, or K^+^ applied at 5 mM did not evoke ion currents (Figures 1B–D and Supplementary Figure 3). At a concentration of 1 μM, strychnine—a well-established antagonist of GlyRs—reversibly inhibited Cs^+^-evoked ion currents through GlyR α3L^185L^ (percent current left: 6.158 ± 1.852%, *n* = 8, *p = 0.039; Figures 1E, F) and the other RNA-edited and unedited GlyR variants investigated here (Supplementary Figure 4), indicating that Cs^+^ had activated ion channels formed by GlyRs. In contrast to Cs^+^, none of the other alkali metals applied at 5 mM had a significant effect on GlyR α3L^185*P*^-expressing HEK293T cells. Thus, it appeared as if the alkali metal ion Cs^+^ at a concentration of 5 mM acted as an agonist for channels formed by RNA-edited GlyR α3L^185L^.

**FIGURE 2:**
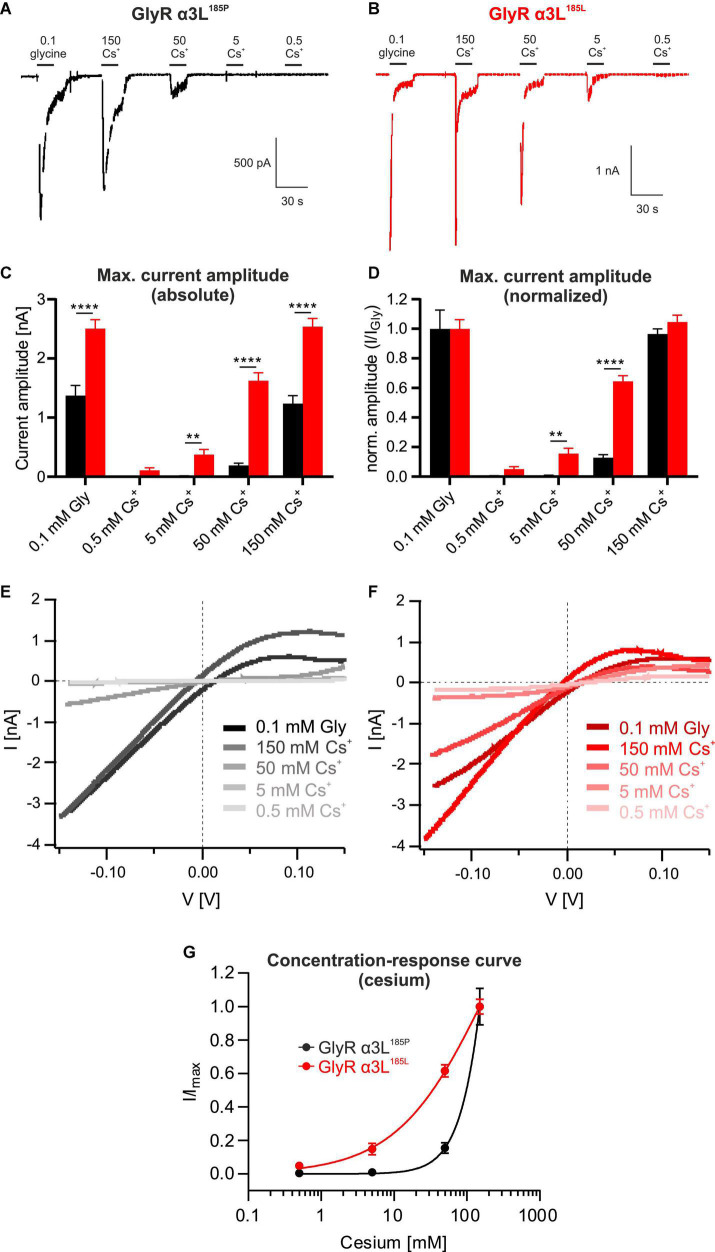
Unedited GlyR α3L^185P^ responds to cesium at higher concentrations. **(A,B)** Representative patch clamp recordings from HEK293T cells expressing GlyR α3L^185P^ [**(A)**, black] or the RNA-edited GlyR α3L^185L^ [**(B)**, red] at a holding potential of −50 mV. Cells were consecutively perfused with 0.1 mM glycine, 0.5 mM Cs^+^, 5 mM Cs^+^, 50 mM Cs^+^, or 150 mM Cs^+^. Scale bars are 30 s horizontal, 500 pA and 1 nA vertical. **(C,D)** Quantification of the absolute **(C)** and glycine response-normalized **(D)** maximum current amplitude of HEK293T cells expressing GlyR α3L^185P^ (black, *n* = 28) or GlyR α3L^185L^ (red, *n* = 23) at the applications shown in **(A,B)**. Data are presented as mean ± SEM. **p < 0.01, ****p < 0.0001. **(E,F)** IV relationships of GlyR α3L^185P^ [**(E)**, *n* = 28] and GlyR α3L^185L^ [**(F)**, *n* = 23] in response to 0.1 mM glycine, 0.5 mM Cs^+^, 5 mM Cs^+^, 50 mM Cs^+^, or 150 mM Cs^+^. Lines indicate means of all cells under the respective condition. All lines were corrected for the liquid junction potentials of the respective solutions (0.1 mM glycine: 10.529 mV; 0.5 mM Cs^+^: 10.485 mV; 5 mM Cs^+^: 10.098 mV; 50 mM Cs^+^: 6.614 mV; 150 mM Cs^+^: 0.618 mV). **(G)** Concentration- response curves describing the current amplitudes for GlyR α3L^185P^ (*n* = 28, black) and GlyR α3L^185L^ (*n* = 23, red) normalized to the highest current responses. Data are presented as mean ± SEM. Non-linear regression was performed using the Hill equation.

To further study Cs^+^-evoked ion currents through GlyR α3L we tested a wider range of Cs^+^ concentrations (Figures 2A, B). The RNA-edited GlyR α3L^185L^ responded to Cs^+^ at concentrations as low as 0.5 mM (0.109 ± 0.041 nA, *n* = 23; Figures 2C, D) and concentration-dependently increased to 0.376 ± 0.085 nA at 5 mM Cs^+^ (*n* = 23, Figures 2C, D), to 1.623 ± 0.135 nA at 50 mM Cs^+^ (*n* = 23, Figures 2C, D) and to 2.539 ± 0.138 nA at 150 mM Cs^+^ (*n* = 23; Figures 2C, D). The concentration-response curve of GlyR α3L^185L^ for Cs^+^ still appeared to be increasing at 150 mM Cs^+^, however, the responses at 150 mM Cs^+^ were similar to those at 0.1 mM glycine (2.539 ± 0.138 nA and 2.503 ± 0.154 nA, respectively; Figures 2C, D), suggesting that the activation of GlyR α3L^185L^ was close to maximum at this concentration. IV relationships (Figures 2E, F) were outwardly rectifying and reversed close to the Nernst potential for Cl^–^ (–3.15 mV), which is compatible with previous data regarding GlyR α3L^185L^ properties of glycine-induced currents (Raltschev et al., 2016). The unedited variant GlyR α3L^185P^ was also activated by Cs^+^, although at higher concentrations. We saw a concentration-dependent activation of GlyR α3L^185P^ beginning at 50 mM and increasing at 150 mM Cs^+^ (0.191 ± 0.038 nA and 1.235 ± 0.135 nA, respectively, *n* = 28; Figures 2A, C, D). At 150 mM Cs^+^, currents had comparable amplitudes to the application of 0.1 mM glycine (1.235 ± 0.135 nA and 1.371 ± 0.174 nA, respectively, *n* = 28; Figures 2A, C, D). Please note that application of 0.1 mM glycine represents a subsaturating condition of GlyR activation, with GlyR α3L^185L^ being more close to saturation than GlyR α3L^185P^ (Legendre et al., 2009). Nevertheless, these results demonstrate considerable activation of the unedited GlyR α3L^185P^ variant by extracellular Cs^+^ concentrations that are still used for open channel blockade of K^+^ channels (Alexander et al., 2019). As for edited GlyR α3L^185L^, Cs^+^-evoked GlyR α3L^185P^ IV relationships were comparable to the glycine-evoked ones, including their shape and outwardly rectifying IV profile (Figures 2E, F). The calculated concentration-response curves using the Hill equation show that RNA-edited GlyR α3L^185L^ is more sensitive in responding to Cs^+^ than the unedited GlyR α3L^185P^ (hill slopes: GlyR α3L^185P^ = 1.793, GlyR α3L^185L^ = 0.703; Figure 2G).

### Cs^+^ activates other RNA variants of GlyR channels

**FIGURE 3:**
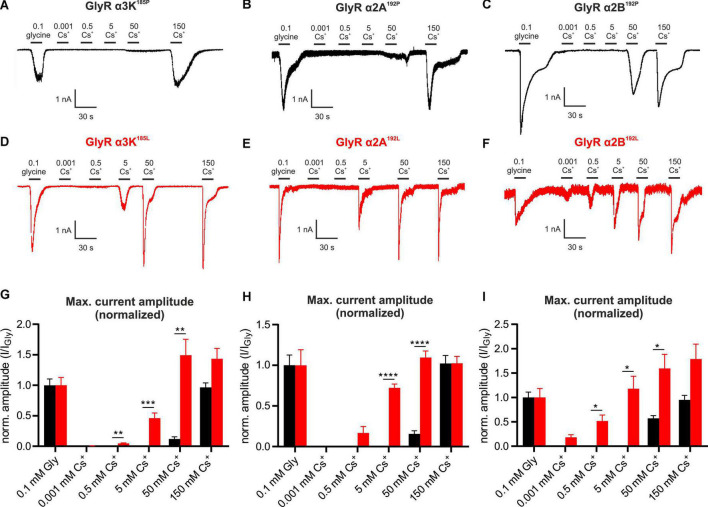
Cesium activates GlyR variants α3K, α2A, and α2B. **(A–F)** Patch clamp recordings from HEK293T cells expressing unedited GlyR α3K^185P^, GlyR α2A^192P^, GlyR α2B^192P^ **(A–C**, black) or RNA-edited GlyR α3K^185L^, GlyR α2A^192L^, GlyR α2B^192L^ [**(D–F)**, red] at a holding potential of –50 mV. Cells were consecutively perfused with 0.1 mM glycine, 0.001 mM Cs^+^, 0.5 mM Cs^+^, 5 mM Cs^+^, 50 mM Cs^+^, or 150 mM Cs^+^. Scale bars are 30 s horizontal, 500 pA and 1 nA vertical. **(G–I)** Quantification of the glycine response-normalized maximum current amplitudes of HEK293T cells expressing GlyR α3K^185P^ [**(G)**, black, *n* = 11] or GlyR α3K^185L^ [**(G)**, *n* = 13, red], GlyR α2A^192P^ [**(H)**, *n* = 21, black], or GlyR α2A^192L^ [**(H)**, *n* = 14, red], GlyR α2B^192P^ [**(I)**, *n* = 15, black] or GlyR α2B^192L^ [**(I)**, *n* = 9, red) perfused with 0.1 mM glycine, 0.001 mM Cs^+^, 0.5 mM Cs^+^, 5 mM Cs^+^, 50 mM Cs^+^, or 150 mM Cs +. Data are presented as mean ± SEM. *p < 0.05, **p < 0.01, ***p < 0.001, ****p < 0.0001.

We tested the effect of Cs^+^ on other GlyR variants, namely the edited and unedited RNA splice variants of GlyR α3K, GlyR α2A and GlyR α2B (Figure 3). Again, each of these subunits were expressed in HEK293T cells to perform whole-cell voltage clamp recordings. Similar to GlyR α3L, the current amplitudes of the edited splice variant GlyR α3K^185L^ to 0.1 mM glycine and to Cs^+^ were significantly higher than the current amplitudes generated by unedited GlyR α3K^185P^ (Figures 3A, D, G and Supplementary Figures 5A, D). Following normalization to glycine-evoked current amplitudes, we detected significantly larger responses from GlyR α3K^185L^ compared to GlyR α3K^185P^ in response to 0.5 mM Cs^+^, 5 mM Cs^+^, and 50 mM Cs^+^ (Figure 3G). The difference was particularly apparent at 5 mM (GlyR α3K^185P^: 0.000 ± 0.000, *n* = 11; GlyR α3K^185L^: 0.460 ± 0.085, *n* = 13; ***p < 0.001; Figure 3G) and 50 mM Cs^+^ (GlyR α3K^185P^: 0.119 ± 0.036, *n* = 11; GlyR α3K^185L^: 1.493 ± 0.261, *n* = 13; **p < 0.01; Figure 3G).

As it was previously reported that RNA-editing of GlyR α2A and GlyR α2B, resulting in P192L amino acid substitution within the mature GlyR polypeptide, increases affinity for glycine (Eichler et al., 2008), we investigated the effect of Cs^+^ on unedited and RNA-edited GlyR α2A and α2B splice variants (Figures 3B, E, H and Figures 3C, F, I, respectively; Supplementary Figure 5). Application of 5 mM Cs^+^ (GlyR α2A^192P^: 0.000 ± 0.000, *n* = 21; GlyR α2A^192L^: 0.722 ± 0.046, *n* = 14; ****p < 0.0001; Figure 3H) and of 50 mM Cs^+^ (GlyR α2A^192P^: 0.156 ± 0.038, *n* = 21; GlyR α2A^192L^: 1.096 ± 0.080, *n* = 14; ****p < 0.0001; Figure 3H) resulted in significantly larger normalized current amplitudes generated by RNA-edited GlyR α2A^192L^ compared to unedited GlyR α2A^192P^.

All tested Cs^+^ concentrations evoked responses from cells expressing the RNA-edited GlyR α2B^192L^ splice variant (Figures 3C, F). Significantly larger normalized current amplitudes compared to the unedited GlyR α2B^192P^ splice variant were observed at 0.5 mM Cs^+^ (GlyR α2B^192P^: 0.000 ± 0.000, *n* = 15; GlyR α2B^192L^: 0.516 ± 0.123, *n* = 9; *p < 0.05; Figure 3I), at 5 mM Cs^+^ (GlyR α2B^192P^: 0.000 ± 0.000, *n* = 15; GlyR α2B^192L^: 1.177 ± 0.256, *n* = 9; *p < 0.05; Figure 3I) as well as at 50 mM Cs^+^ (GlyR α2B^192P^: 0.570 ± 0.056, *n* = 15; GlyR α2B^192L^: 1.595 ± 0.289, *n* = 9; *p < 0.05; Figure 3I).

The calculated concentration-response curves using the Hill equation show that RNA-edited GlyR α3K^185L^, GlyR α2A^192L^, and GlyR α2B^192L^ are more sensitive in responding to Cs^+^ than their unedited GlyR variants (hill slopes: GlyR α3K^185L^ = 1.561 and GlyR α3K^185P^ = 1.924; GlyR α2A^192L^ = 1.080 and GlyR α2A^192P^ = 1.894; GlyR α2B^192L^ = 0.599 and GlyR α2B^192P^ = 7.531; Supplementary Figures 5D–F).

As described earlier (Kletke et al., 2013; Winkelmann et al., 2014), GlyRs—and especially RNA-edited GlyR variants—show basal activity in the absence of an agonist, explaining the relatively high noise levels in some recording traces. Indeed, noise can be reduced when applying 1 μM strychnine (Supplementary Figure 6).

In summary, these results identify Cs^+^ as agonist of both unedited and RNA-edited GlyR α2A, α2B, α3K, and α3L RNA splice variants. RNA-edited GlyR variants responded in a more sensitive way, as was the case for all agonists tested so far—including taurine, GABA, and NH_4_^+^ (Meier et al., 2005; Legendre et al., 2009; Kankowski et al., 2018).

### Computational analysis of GlyR binding sites for monovalent cations

**FIGURE 4:**
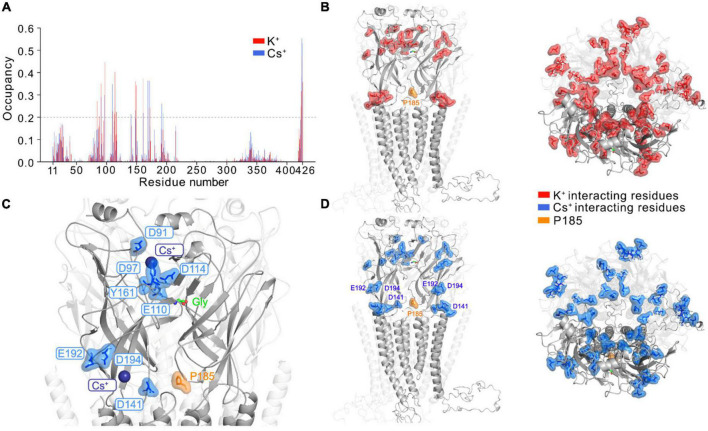
Monovalent cation binding sites in GlyR α3 identified from MD simulations. **(A)** The time-averaged residue-wise ion occupancy calculated from Cs^+^ (blue) and K^+^ (red) simulations, respectively. At each snapshot, a residue was considered to interact with an ion if the distance between the residue and ion is smaller than the hydration radius of the ion (3.4 Å for K^+^ and 3.8 Å for Cs^+^). The side- (left) and top-view (right) of the GlyR α3 with main ion interacting residues (ion occupancy larger than 0.2) highlighted for **(B)** K^+^ (red) and **(D)** Cs^+^ (blue), respectively. For clarity, one subunit of the pentamer in front is highlighted, and the editable site P185 is shown in orange. The MD simulations were only performed with the unedited variant P185. **(C)** Two major binding sites for Cs^+^ at the extracellular domain: (i) slightly above the glycine binding pocket; (ii) in the neighborhood of the editable site P185. The protein is shown in cartoon, glycine as green sticks, editable site P185 as orange sticks, Cs^+^ as blue sphere and key interacting residues of Cs^+^ are labeled and shown as blue sticks.

We investigated the possible mechanism for GlyR activation by Cs^+^ by performing atomistic MD simulations of full-length GlyR protein embedded in a membrane bilayer with K^+^ and Cs^+^, respectively. Starting with the open conformation of GlyR α3K (homology structure of GlyR α1 determined by cryo-EM, PDB ID: 6UD3, Kumar et al., 2020), we removed glycine from its binding pocket and performed the simulations with unedited GlyR α3K in its apo form using three different force field and water model combinations: (i) Charmm36m + TIP3P; (ii) Amber19SB + TIP3P; (iii) Amber19SB + OPC. For each simulation setup, we calculated the time-averaged ion occupancy residue-wise from three runs of 300 ns simulations for K^+^ and Cs^+^, respectively (Figure 4A and Supplementary Figure 7A). We considered a residue to be interacting with an ion if the distance between the two was smaller than the hydration radius of the ion (3.4 Å for K^+^ and 3.8 Å for Cs^+^, Caralampio et al., 2017; Biedermann et al., 2021). During the MD simulations at the current time scale, we rarely observed ions binding to all sites simultaneously. Therefore, a binding event was considered to occur when the corresponding residue from at least one subunit interacted with the ions. The results summarized in Figures 4A, B, D showed that the majority of protein-cation contacts were found for the residues in the extracellular domain of the GlyR α3K, with only a few interactions observed in the transmembrane and intracellular domains. This observation may be explained by the computation of the electrostatic surface of the GlyR α3K (Supplementary Figure 7C), which revealed a strong negative potential in the extracellular domain for interacting with cations. In contrast, a strong positive potential was computed for the transmembrane region, where the chloride ions traverse the channel. Furthermore, the simulations revealed highly similar protein-ion binding profiles for K^+^ and Cs^+^ (Figures 4A, B, D). Although the residue-wise absolute Cs^+^ occupancy vary in the simulations with different force field and water model combinations, the overall trend is almost indistinguishable (Supplementary Figure 7A). There are two regions in the extracellular domain that showed significant monovalent cation binding: (i) above the binding pocket of glycine, where several charged residues including D91, D97, E110, D114 mainly contribute to the interaction with monovalent cations; (ii) around the RNA-edited position P185, where several residues such as D141, E192, D194, showed considerably more pronounced interactions with Cs^+^ compared to K^+^ (Figures 4A, C and Supplementary Movie 1). As ligand residence times are often strongly correlated with ligand binding affinity, we also calculated and compared the residence time for K^+^ and Cs^+^ at the glycine binding pocket. The results, summarized in Supplementary Figure 7B suggested that the binding events were mostly transient and fast, with a few events occurring on the hundred nanosecond time scale.

## Discussion

Cesium is used in electrophysiology as a blocker of voltage-gated potassium channels and hyperpolarization-activated cation currents (Thoby-Brisson et al., 2000; Rateau and Ropert, 2006). Cesium chloride is also advertised as an alternative therapy for different types of cancer. However, it turned out that prolonged self-administration of cesium chloride leads to a severe decline in health, e.g., development of long QT syndrome and ventricular tachycardia, and did not prevent tumor progression (Dalal et al., 2004), associated with undesirable side effects in breast cancer (Sessions et al., 2013). In fact, earlier work already pointed to GlyRs as a target of extracellular Cs^+^ (Lewis et al., 1989; Smith and McBurney, 1989). In this study, we confirm previous findings and provide a more detailed analysis of the different RNA splice and editing variants of GlyR α2 and α3 channels. Together with previous studies, the present work might help to explain the multifaceted effects and actions of cesium in the human body, including effects on CNS and cardiovascular functions.

When ingested, the absorption of cesium chloride is nearly complete and it gets evenly distributed with higher concentrations in the liver, kidney, skeletal muscle, and brain (Centeno et al., 2003; Melnikov and Zanoni, 2010). The excretion is mainly via the kidneys, and the mean long-term biological half-life is ca. 70 days (Iinuma et al., 1965; Melnikov and Zanoni, 2010). For example, high cesium chloride intake (6 g/day) as described in the studies about cesium administration against cancer could theoretically lead to body concentrations of 5 mM Cs^+^ after 2–6 days. The calculation would thereby consider either 40 L body fluid or 12% of total Cs^+^ in a 1.4 kg brain with complete absorption and minimal excretion (Brewer, 1984; Leggett et al., 2003). Upon self-administration, the concentration that would considerably activate RNA-edited GlyRs can therefore be achieved and explain adverse diverse effects of a high cesium chloride diet. The FDA recently published alerts regarding cesium diet (U.S. Food and Drug Administration, 2018, 2020).

In this study, we also investigated other monovalent cations of the alkali metal ion family. However, the data identify only cesium as agonist that activates RNA-edited and unedited GlyRs at millimolar concentrations, while other monovalent alkali metal ions did not exert an agonistic effect on GlyRs. The results show that Cs^+^-evoked responses are GlyR-mediated since co-application of 1 μM strychnine—an established antagonist of GlyR activation—reversibly reduced cesium-dependent current responses through the transfected GlyR variants. Analysis of the different GlyR channel variants arising through RNA editing and splicing (GlyR α2A, GlyR α2B, GlyR α3K, and GlyR α3L) revealed that Cs^+^ activated the C-to-U RNA-edited versions of these receptors at much lower concentrations than their unedited counterparts. C-to-U RNA-editing of GlyR-coding gene transcripts results in gain-of-function GlyR variants (Meier et al., 2005; Legendre et al., 2009), and their expression is elevated in patients with a severe course of TLE (Eichler et al., 2008)—a fact that could add also these persons to the list of patients suffering from cesium susceptibility.

To probe alkali metal binding sites in the full-length GlyR α3K, we conducted atomistic MD simulations with K^+^ and Cs^+^, respectively, using various force field and water model combinations. Previous cryo-EM structures of GlyRs failed to reveal binding sites for monovalent cations of the alkali metal family (Du et al., 2015; Kumar et al., 2020; Yu et al., 2021), possibly due to the low resolution of the determined structures. Our atomistic MD simulations predicted here two pronounced binding sites for Cs^+^ and K^+^ at the extracellular domain of the GlyR: (i) slightly above the glycine binding pocket, which contains several charged residues including D91, D97, E110, D114 that coordinate Cs^+^ and K^+^; and (ii) in the neighborhood of the editable P185 protein site, where several residues (D141, E192, D194) showed considerably more pronounced interactions with Cs^+^ compared to K^+^. We noted that in a previously determined X-ray structure of an acid sensing ion channel (ASIC) (Gonzales et al., 2009), two main Cs^+^ binding sites were also found to be located at the extracellular domain, one of which is close to the putative proton-binding sites. Although our MD simulations performed under the hundred nanosecond timescale cannot resolve the mechanism of Cs^+^-dependent GlyR α3 activation, based on the location of the alkali metal binding sites, two functional consequences of Cs^+^ binding can be considered: (i) As predicted Cs^+^ binding sites and residues of the glycine binding pocket are close to each other, Cs^+^ may stabilize the open chloride-conductive conformation of GlyRs; (ii) Cs^+^ binding close to the editable P185 site may also contribute to stabilization of the open channel configuration and spontaneous activity of the edited form of GlyR α3K, but as of now, the simulations have only been conducted with the unedited variant, for which the structure was resolved.

## Data availability statement

The raw data supporting the conclusions of this article will be made available by the authors, without undue reservation.

## Author contributions

SF, FH, JL, HL, MH, AE, and PK performed experiments. SF, FH, MH, HS, MS, and JM wrote the manuscript or contributed to writing and revision. All authors contributed to the article and approved the submitted version.

## Supplementary material

Supplementary Figure 1 GlyR α3K without HA tag shows similar cesium-induced activation compared to HA-tagged GlyR α3K. **(A,B)** Recordings of transfected HEK293T cells showing the current changes of GlyR α3K^185P^ **(A)** and GlyR α3K^185L^ **(B)** without HA tag in response to 5 mM Cs^+^, 50 mM Cs^+^, and 150 mM Cs^+^. Scale bars are 0.4 nA vertical and 100 s horizontal **(A)** and 1 nA vertical and 50 s horizontal **(B)**.

Supplementary Figure 2 NMR spectroscopy. An overlay of the 1D-^1^H spectra of the 150 mM CsCl, 1M CsOH 1H_2_O, and 150 mM LiCl samples, which were used in the electrophysiology measurements. Glycine samples at different concentrations were used as references. The peak at around 3.5 ppm arises from the CH_2_ protons of the glycine. No signal at the same position was observed for CsCl, CsOH, and LiCl, demonstrating that these samples were not contaminated with glycine.

Supplementary Figure 3 Lithium does not activate unedited or RNA-edited GlyR α3L. Representative patch clamp recordings from HEK293T cells expressing unedited GlyR α3L^185P^ [**(A)**, black] or RNA-edited GlyR α3L^185L^ [**(B)**, red] consecutively perfused with 0.001 mM Li^+^, 0.5 mM Li^+^, 5 mM Li^+^, 50 mM Li^+^, 150 mM Li^+^, or 0.1 mM glycine. Scale bars are 0.5 nA vertical and 25 s horizontal **(A)** and 1 nA vertical and 25 s horizontal **(B)**. **(C)** Quantification of the maximum current amplitude of HEK293T cells expressing GlyR α3L^185P^ (black, *n* = 10) or GlyR α3L^185L^ (red, *n* = 14) perfused with 0.001 mM Li^+^, 0.5 mM Li^+^, 5 mM Li^+^, 50 mM Li^+^, 150 mM Li^+^, or 0.1 mM glycine. Data are presented as mean ± SEM. ****p* < 0.001.

Supplementary Figure 4 Strychnine inhibits GlyR currents evoked by 5 mM and 150 mM cesium. **(A,B)** Patch clamp recordings from HEK293T cells expressing either unedited GlyR α3L^185P^ **(A)** or RNA-edited GlyR α3L^185L^ **(B)** and co-treated with 150 mM Cs^+^ and 1 μM strychnine **(A)** or 5 mM Cs^+^ and 1 μM strychnine **(B)**. Dashed lines indicate 0 nA. Scale bars are 0.2 nA vertical and 20 s **(A)** and 50 s horizontal **(B)**. **(C,D)** Patch clamp recordings from HEK293T cells expressing either GlyR α3K^185P^ **(C)** or GlyR α3K^185L^ **(D)** and co-treated with 150 mM Cs^+^ and 1 μM strychnine **(C)** or 5 mM Cs^+^ and 1 μM strychnine **(D)**. Dashed lines indicate 0 nA. Scale bars are 0.5 nA vertical and 100 s horizontal **(C)** and 0.2 nA vertical and 50 s horizontal **(D)**. **(E,F)** Recordings of HEK293T cells expressing either GlyR α2A^192P^ **(E)** or GlyR α2A^192L^ **(F)** and co-treated with 50 mM Cs^+^ and 1 μM strychnine **(E)** or 5 mM Cs^+^ and 1 μM strychnine **(F)**. Dashed lines indicate 0 nA. Scale bars are 50 s horizontal and 0.2 nA **(E)** and 0.4 nA vertical **(F)**. **(G,H)** Recordings of HEK293T cells expressing either GlyR α2B^192P^ **(G)** or GlyR α2B^192L^ **(H)** and co-treated with 50 mM Cs^+^ and 1 μM strychnine **(G)** or 5 mM Cs^+^ and 1 μM strychnine **(H)**. Dashed lines indicate 0 nA. Scale bars are 50 s horizontal and 0.4 nA **(G)** and 0.1 nA vertical **(H)**.

Supplementary Figure 5 Cesium evokes currents through the GlyR variants α3K, α2A, and α2B. **(A–C)** Quantification of the absolute maximum current amplitude of HEK293T cells expressing GlyR α3K^185P^ (*n* = 11, black) or GlyR α3K^185L^ [**(A)**, *n* = 13, red], GlyR α2A^192P^ (*n* = 21, black) or GlyR α2A^192L^ [**(B)**, *n* = 14, red], GlyR α2B^192P^ (*n* = 15, black) or GlyR α2B^192L^ [**(C)**, *n* = 9, red] perfused with 0.1 mM glycine, 0.001 mM Cs^+^, 0.5 mM Cs^+^, 5 mM Cs^+^, 50 mM Cs^+^, or 150 mM Cs^+^. Data are presented as mean ± SEM. **p* < 0.05, ***p* < 0.01, ****p* < 0.001, *****p* < 0.0001. **(D–F)** Concentration-response curves describing the current amplitudes for GlyR α3K^185P^ [**(D)**, *n* = 11, black] and GlyR α3K^185L^ [**(D)**, *n* = 13, red], GlyR α2A^192P^ [**(E)**, *n* = 21, black], and GlyR^192L^ [**(E)**, *n* = 14, red] as well as GlyR α2B^192P^ [**(F)**, *n* = 15, black] and GlyR α2B^192L^ [**(F)**, *n* = 9, red] normalized to the highest current responses, respectively. Data are presented as mean ± SEM. Non-linear regression was performed using the Hill equation. **(G)** Recording of an untransfected HEK293T cell perfused with 150 mM Cs^+^ at two different time points for 30 s. Dashed line indicates 0 nA. Scale bar is 0.2 nA vertical and 20 s horizontal. **(H,I)** Recordings of either GlyR α3L^185P^ **(H)** or GlyR α3L^185L^ **(I)** expressing HEK293T cells treated with 150 mM NaCl followed by 150 mM NMDG^+^. Dashed lines indicate 0 pA. Scale bars are 5 s horizontal as well as 0.5 nA **(I)** and 0.4 nA vertical **(I)**.

Supplementary Figure 6 Basal activity of RNA-edited GlyRs is reduced by strychnine. **(A–C)** Recordings of HEK293T cells expressing GlyR α3K^185L^ **(A)**, GlyR α2A^192L^ **(B)**, or GlyR α2B^185L^ **(C)** treated with 1 μM strychnine in the absence of an agonist. Dashed lines indicate 0 nA. Scale bars are 0.2 nA vertical and 40 s horizontal **(A)**, 0.1 nA vertical, and 50 s horizontal **(B)**, and 0.2 nA vertical and 20 s horizontal **(C)**.

Supplementary Figure 7 K^+^ and Cs^+^ binding sites determined from MD with different force field and water model combinations. **(A)** The time-averaged residue-wise Cs^+^ occupancy calculated from simulations with three different force field and water model combinations. At each snapshot, a residue is considered to interact with an ion if the distance between the residue and ion is smaller than the hydration radius of the ion (3.8 Å for Cs^+^). **(B)** The distributions of residence time for K^+^ and Cs^+^ at the major binding site that were derived from MD simulations. Binding was considered if the distance between Cs^+^ and at least two residues at the binding site was within a 7 Å sphere. (C, left) The sectional view of GlyR α3 shown as electrostatic potential surface. Red and blue surfaces represent negative and positive electrostatic potential, respectively. (Right) A selected snapshot of GlyR α3 from MD simulations shows that, due to differences in the electrostatic potential between the extracellular and transmembrane domains, Cs^+^ (cyan) and Cl^–^ (green) were mainly distributed in the extracellular and transmembrane parts of GlyR α3, respectively.

## References

[B1] Alexander S. P. H. Mathie A. Peters J. A. Veale E. L. Striessnig J. Kelly E. (2019). The concise guide to pharmacology 2019/20: ion channels. *Br. J. Pharmacol.* 176 Suppl 1 (Suppl. 1), S142–S228. 31710715 10.1111/bph.14749 PMC6844578

[B2] Betz H. Laube B. (2006). Glycine receptors: recent insights into their structural organization and functional diversity. *J. Neurochem*. 97 1600–1610. 10.1111/j.1471-4159.2006.03908.x 16805771

[B3] Biedermann J. Braunbeck S. Plested A. J. R. Sun H. (2021). Nonselective cation permeation in an AMPA-type glutamate receptor. *Proc. Natl. Acad. Sci. U S A.* 118:e2012843118. 10.1073/pnas.2012843118 33602810 PMC7923540

[B4] Brewer A. K. (1984). The high pH therapy for cancer tests on mice and humans. *Pharmacol. Biochem. Behav.* 21 (Suppl. 1), 1–5. 10.1016/0091-3057(84)90152-7 6522424

[B5] Bussi G. Donadio D. Parrinello M. (2007). Canonical sampling through velocity rescaling. *J. Chem. Phys.* 126:14101. 10.1063/1.2408420 17212484

[B6] Çaliskan G. Müller I. Semtner M. Winkelmann A. Raza A. S. Hollnagel J. O. (2016). Identification of parvalbumin interneurons as cellular substrate of fear memory persistence. *Cereb. Cortex* 26 2325–2340. 10.1093/cercor/bhw001 26908632 PMC4830301

[B7] Caralampio D. Z. Martínez J. M. Pappalardo R. R. Marcos E. S. (2017). The hydration structure of the heavy-alkalines Rb+ and Cs+ through molecular dynamics and X-ray absorption spectroscopy: surface clusters and eccentricity. *Phys. Chem. Chem. Phys.* 19 28993–29004. 10.1039/c7cp05346k 29063078

[B8] Centeno J. A. Pestaner J. P. Omalu B. I. Torres N. L. Field F. Wagner G. (2003). Blood and tissue concentration of cesium after exposure to cesium chloride: a report of two cases. *Biol. Trace Element Res.* 94 97–104. 10.1385/BTER:94:2:97 12958400

[B9] Clay J. R. Shlesinger M. F. (1984). Analysis of the effects of cesium ions on potassium channel currents in biological membranes. *J. Theor. Biol.* 107 189–201. 6325824 10.1016/s0022-5193(84)80021-1

[B10] Dalal A. K. Harding J. D. Verdino R. J. (2004). Acquired long QT syndrome and monomorphic ventricular tachycardia after alternative treatment with cesium chloride for brain cancer. *Mayo Clinic Proceed.* 79 1065–1069. 10.4065/79.8.1065 15301336

[B11] Darden T. York D. Pedersen L. (1993). Particle mesh Ewald: an N log(N) method for Ewald sums in large systems. *J. Chem. Phys.* 98 10089–10092.

[B12] DiFrancesco D. (1982). Block and activation of the pace-maker channel in calf purkinje fibres: effects of potassium, caesium and rubidium. *J. Physiol.* 329 485–507. 10.1113/jphysiol.1982.sp014315 6292407 PMC1224792

[B13] Du J. Lü W. Wu S. Cheng Y. Gouaux E. (2015). Glycine receptor mechanism elucidated by electron cryo-microscopy. *Nature* 526 224–229. 10.1038/nature14853 26344198 PMC4659708

[B14] Eichler S. A. Förstera B. Smolinsky B. Jüttner R. Lehmann T.-N. Fähling M. (2009). Splice-specific roles of glycine receptor alpha3 in the hippocampus. *Eur. J. Neurosci.* 30 1077–1091. 10.1111/j.1460-9568.2009.06903.x 19723286

[B15] Eichler S. A. Kirischuk S. Jüttner R. Schaefermeier P. K. Schafermeier P. K. Legendre P. (2008). Glycinergic tonic inhibition of hippocampal neurons with depolarizing GABAergic transmission elicits histopathological signs of temporal lobe epilepsy. *J. Cell. Mol. Med.* 12 2848–2866. 10.1111/j.1582-4934.2008.00357.x 19210758 PMC3828897

[B16] Förstera B. Dzaye O. Winkelmann A. Semtner M. Benedetti B. Markovic D. S. (2014). Intracellular glycine receptor function facilitates glioma formation in vivo. *J. Cell Sci.* 127(Pt 17), 3687–3698. 10.1242/jcs.146662 24994934

[B17] Gonzales E. B. Kawate T. Gouaux E. (2009). Pore architecture and ion sites in acid-sensing ion channels and P2X receptors. *Nature* 460 599–604. 10.1038/nature08218 19641589 PMC2845979

[B18] Harvey R. J. Depner U. B. Wässle H. Ahmadi S. Heindl C. Reinold H. (2004). GlyR alpha3: an essential target for spinal PGE2-mediated inflammatory pain sensitization. *Science* 304 884–887. 10.1126/science.1094925 15131310

[B19] Hess B. Bekker H. Berendsen H. J. C. Fraaije J. G. E. M. (1997). LINCS: a linear constraint solver for molecular simulations. *J. Comput. Chem.* 18 1463–1472.

[B20] Huang J. Rauscher S. Nawrocki G. Ran T. Feig M. Groot B. L. (2016). CHARMM36m: an improved force field for folded and intrinsically disordered proteins. *Nat. Methods* 14 71–73. 27819658 10.1038/nmeth.4067 PMC5199616

[B21] Iinuma T. Nagai T. Ishihara T. Watari K. Izawa M. (1965). Cesium turnover in man following single administration of 132 Cs. i. whole body retention and excretion pattern. *J. Radiation Res.* 6 73–81. 10.1269/jrr.6.73 5883137

[B22] Izadi S. Anandakrishnan R. Onufriev A. V. (2014). Building water models: A different approach. *J. Phys. Chem. Lett*. 5, 3863–3871. 10.1021/jz501780a 25400877 PMC4226301

[B23] Jo S. Kim T. Iyer V. G. Im W. (2008). CHARMM-GUI: a web-based graphical user interface for CHARMM. *J. Comput. Chem.* 29 1859–1865. 18351591 10.1002/jcc.20945

[B24] Jorgensen W. L. Chandrasekhar J. Madura J. D. Impey R. W. Klein M. L. (1983). Comparison of simple potential functions for simulating liquid water. *J. Chem. Phys.* 79 926–935.

[B25] Kankowski S. Förstera B. Winkelmann A. Knauff P. Wanker E. E. You X. A. (2018). A novel RNA editing sensor tool and a specific agonist determine neuronal protein expression of RNA-edited glycine receptors and identify a genomic APOBEC1 dimorphism as a new genetic risk factor of epilepsy. *Front. Mol. Neurosci.* 10:439. 10.3389/fnmol.2017.00439 29375302 PMC5768626

[B26] Kletke O. Sergeeva O. A. Lorenz P. Oberland S. Meier J. C. Hatt H. (2013). New insights in endogenous modulation of ligand-gated ion channels: histamine is an inverse agonist at strychnine sensitive glycine receptors. *Eur. J. Pharmacol.* 710 59–66. 10.1016/j.ejphar.2013.04.002 23603522

[B27] Kumar A. Basak S. Rao S. Gicheru Y. Mayer M. L. Sansom M. S. P. (2020). Mechanisms of activation and desensitization of full-length glycine receptor in lipid nanodiscs. *Nat. Commun.* 11:3752. 10.1038/s41467-020-17364-5 32719334 PMC7385131

[B28] Leacock S. Syed P. James V. M. Bode A. Kawakami K. Keramidas A. (2018). Structure/function studies of the α4 subunit reveal evolutionary loss of a GlyR subtype involved in startle and escape responses. *Front. Mol. Neurosci.* 11:23. 10.3389/fnmol.2018.00023 29445326 PMC5797729

[B29] Lee J. Cheng X. Swails J. M. Yeom M. S. Eastman P. K. Lemkul J. A. (2016). CHARMM-GUI input generator for NAMD, GROMACS, AMBER, OpenMM, and CHARMM/OpenMM simulations using the CHARMM36 additive force field. *J. Chem. Theory Comput.* 12 405–413. 10.1021/acs.jctc.5b00935 26631602 PMC4712441

[B30] Legendre P. (2001). The glycinergic inhibitory synapse. *Cell. Mol. Life Sci.* 58 760–793. 11437237 10.1007/PL00000899 PMC11337367

[B31] Legendre P. Förstera B. Jüttner R. Meier J. C. (2009). Glycine receptors caught between genome and proteome – functional implications of RNA editing and splicing. *Front. Mol. Neurosci.* 2:23. 10.3389/neuro.02.023.2009 19936314 PMC2779093

[B32] Leggett R. W. Williams L. R. Melo D. R. Lipsztein J. L. (2003). A physiologically based biokinetic model for cesium in the human body. *Sci. Total Environ.* 317 235–255. 10.1016/S0048-9697(03)00333-4 14630424

[B33] Lemmens V. Thevelein B. Vella Y. Kankowski S. Leonhard J. Mizuno H. (2022). Hetero-pentamerization determines mobility and conductance of Glycine receptor α3 splice variants. *Cell. Mol. Life Sci.* 79:540. 10.1007/s00018-022-04506-9 PMC9534812 36197517

[B34] Lewis C. A. Ahmed Z. Faber D. S. (1989). Characteristics of glycine-activated conductances in cultured medullary neurons from embryonic rat. *Neurosci. Lett.* 96 185–190. 10.1016/0304-3940(89)90055-4 2538783

[B35] McCracken L. M. Lowes D. C. Salling M. C. Carreau-Vollmer C. Odean N. N. Blednov Y. A. (2017). Glycine receptor α3 and α2 subunits mediate tonic and exogenous agonist-induced currents in forebrain. *Proc. Natl. Acad. Sci. U S A.* 114 E7179–E7186. 28784756 10.1073/pnas.1703839114 PMC5576794

[B36] Meier J. Vannier C. Sergé A. Triller A. Choquet D. (2001). Fast and reversible trapping of surface glycine receptors by gephyrin. *Nat. Neurosci.* 4 253–260. 10.1038/85099 11224541

[B37] Meier J. C. Henneberger C. Melnick I. Racca C. Harvey R. J. Heinemann U. (2005). RNA editing produces glycine receptor alpha3(P185L), resulting in high agonist potency. *Nat. Neurosci.* 8 736–744. 10.1038/nn1467 15895087

[B38] Meier J. C. Semtner M. Winkelmann A. Wolfart J. (2014). Presynaptic mechanisms of neuronal plasticity and their role in epilepsy. *Front. Cell. Neurosci.* 8:164. 10.3389/fncel.2014.00164 24987332 PMC4060558

[B39] Melnikov P. Zanoni L. Z. (2010). Clinical effects of cesium intake. *Biol. Trace Element Res.* 135 1–9. 10.1007/s12011-009-8486-7 19655100

[B40] Meyer G. Kirsch J. Betz H. Langosch D. (1995). Identification of a gephyrin binding motif on the glycine receptor beta subunit. *Neuron* 15 563–572. 7546736 10.1016/0896-6273(95)90145-0

[B41] Miller P. S. Harvey R. J. Smart T. G. (2004). Differential agonist sensitivity of glycine receptor alpha2 subunit splice variants. *Br. J. Pharmacol.* 143 19–26. 10.1038/sj.bjp.0705875 15302677 PMC1575261

[B42] Nikolic Z. Laube B. Weber R. G. Lichter P. Kioschis P. Poustka A. (1998). The human glycine receptor subunit alpha3. Glra3 gene structure, chromosomal localization, and functional characterization of alternative transcripts. *J. Biol. Chem.* 273 19708–19714. 10.1074/jbc.273.31.19708 9677400

[B43] Notelaers K. Rocha S. Paesen R. Smisdom N. Clercq B. Meier J. C. (2014a). Analysis of alpha3 GlyR single particle tracking in the cell membrane. *Biochimica Biophys. Acta* 1843 544–553. 10.1016/j.bbamcr.2013.11.019 24316136

[B44] Notelaers K. Rocha S. Paesen R. Swinnen N. Vangindertael J. Meier J. C. (2014b). Membrane distribution of the glycine receptor α3 studied by optical super-resolution microscopy. *Histochem. Cell Biol.* 142 79–90. 24553792 10.1007/s00418-014-1197-y

[B45] Notelaers K. Smisdom N. Rocha S. Janssen D. Meier J. C. Rigo J.-M. (2012). Ensemble and single particle fluorimetric techniques in concerted action to study the diffusion and aggregation of the glycine receptor α3 isoforms in the cell plasma membrane. *Biochim. Biophys. Acta* 1818 3131–3140. 22906711 10.1016/j.bbamem.2012.08.010

[B46] Parrinello M. Rahman A. (1981). Polymorphic transitions in single crystals: a new molecular dynamics method. *J. Appl. Phys.* 52 7182–7190. 10.1039/c9dt02916h 31552956

[B47] Raltschev C. Hetsch F. Winkelmann A. Meier J. C. Semtner M. (2016). Electrophysiological signature of homomeric and heteromeric glycine receptor channels. *J. Biol. Chem.* 291 18030–18040. 10.1074/jbc.M116.735084 27382060 PMC5016189

[B48] Rateau Y. Ropert N. (2006). Expression of a functional hyperpolarization-activated current (Ih) in the mouse nucleus reticularis thalami. *J. Neurophysiol.* 95 3073–3085. 10.1152/jn.00922.2005 16617177

[B49] Schewe M. Nematian-Ardestani E. Sun H. Musinszki M. Cordeiro S. Bucci G. (2016). A non-canonical voltage-sensing mechanism controls gating in K2P K(+) channels. *Cell* 164 937–949. 10.1016/j.cell.2016.02.002 26919430 PMC4771873

[B50] Schindelin J. Arganda-Carreras I. Frise E. Kaynig V. Longair M. Pietzsch T. (2012). Fiji: an open-source platform for biological-image analysis. *Nat. Methods* 9 676–682. 10.1038/nmeth.2019 22743772 PMC3855844

[B51] Sessions D. Heard K. Kosnett M. (2013). Fatal cesium chloride toxicity after alternative cancer treatment. *J. Alternative Complementary Med.* 19 973–975. 23841836 10.1089/acm.2012.0745 PMC3868249

[B52] Smith S. M. McBurney R. N. (1989). Caesium ions: a glycine-activated channel agonist in rat spinal cord neurones grown in cell culture. *Br. J. Pharmacol.* 96 940–948. 10.1111/j.1476-5381.1989.tb11905.x 2472848 PMC1854446

[B53] Thoby-Brisson M. Telgkamp P. Ramirez J.-M. (2000). The role of the hyperpolarization-activated current in modulating rhythmic activity in the isolated respiratory network of mice. *J. Neurosci.* 20 2994–3005. 10.1523/JNEUROSCI.20-08-02994.2000 10751452 PMC6772196

[B54] Tian C. Kasavajhala K. Belfon K. Raguette L. Huang H. Migues A. (2020). ff19SB: Amino-acid-specific protein backbone parameters trained against quantum mechanics energy surfaces in solution. *J. Chem. Theory Comput*. 16, 528–552. 10.1021/acs.jctc.9b00591 31714766 PMC13071887

[B55] U.S. Food and Drug Administration (2018). FDA Alerts Health Care Professionals of Significant Safety Risks Associated with Cesium Chloride [Press Release]. Available online at: https://www.fda.gov/drugs/human-drug-compounding/fda-alerts-health-care-professionals-significant-safety-risks-associated-cesium-chloride (accessed February 2023).

[B56] U.S. Food and Drug Administration (2020). *Public Health Alert Concerning Dietary Supplements Containing Cesium Salts [Press Release].* Available online at https://www.fda.gov/food/dietary-supplement-products-ingredients/public-health-alert-concerning-dietary-supplements-containing-cesium-salts (accessed February 2023).

[B57] Waterhouse A. Bertoni M. Bienert S. Studer G. Tauriello G. Gumienny R. (2018). SWISS-MODEL: homology modelling of protein structures and complexes. *Nucleic Acids Res.* 46 W296–W303. 29788355 10.1093/nar/gky427 PMC6030848

[B58] Winkelmann A. Maggio N. Eller J. Caliskan G. Semtner M. Häussler U. (2014). Changes in neural network homeostasis trigger neuropsychiatric symptoms. *J. Clin. Investigation* 124 696–711. 10.1172/JCI71472 24430185 PMC3904623

[B59] Winkelmann A. Semtner M. Meier J. C. (2015). Chloride transporter KCC2-dependent neuroprotection depends on the N-terminal protein domain. *Cell Death Dis.* 6:e1776. 10.1038/cddis.2015.127 26043076 PMC4669822

[B60] Yu J. Zhu H. Lape R. Greiner T. Du J. Lü W. (2021). Mechanism of gating and partial agonist action in the glycine receptor. *Cell* 184 957–968.e21. 33567265 10.1016/j.cell.2021.01.026 PMC8115384

