# Supplementary material for: Cesium activates the neurotransmitter receptor for glycine
Source: Front Mol Neurosci. 2023 May 22;16:1018530. doi: 10.3389/fnmol.2023.1018530 (PMC10239821; doi:10.3389/fnmol.2023.1018530)
Supplement: Supplementary Figure 1 — GlyR α3K without HA tag shows similar cesium-induced activation compared to HA-tagged GlyR α3K. (A,B) Recordings of transfected HEK293T cells showing the current changes of GlyR α3K185P (A) and GlyR α3K185L (B) without HA tag in response to 5 mM Cs+, 50 mM Cs+, and 150 mM Cs+. Scale bars are 0.4 nA vertical and 100 s horizontal (A) and 1 nA vertical and 50 s horizontal (B). [file Presentation_1.pptx]

## Slide 1
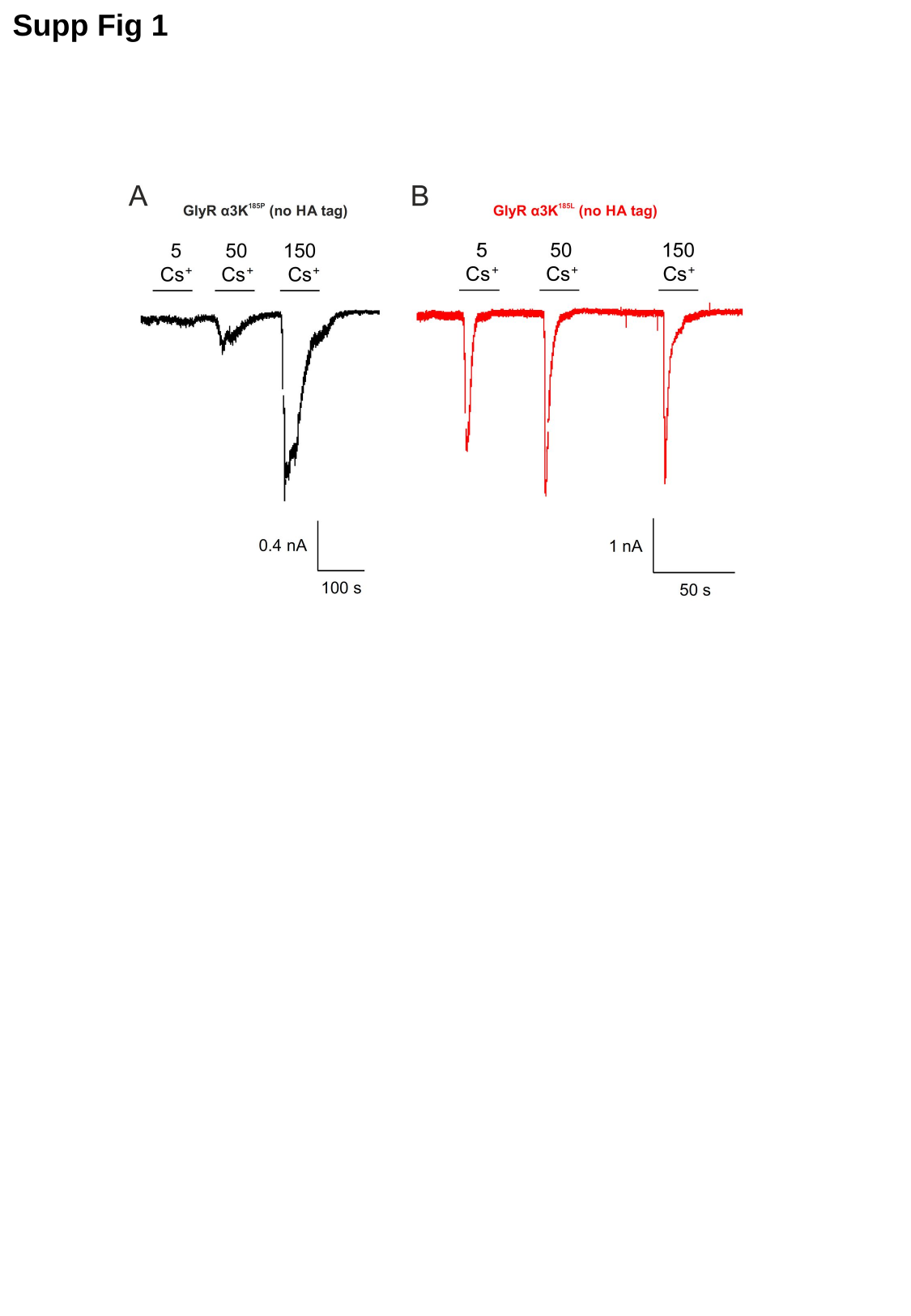

Supp Fig 1

## Slide 2
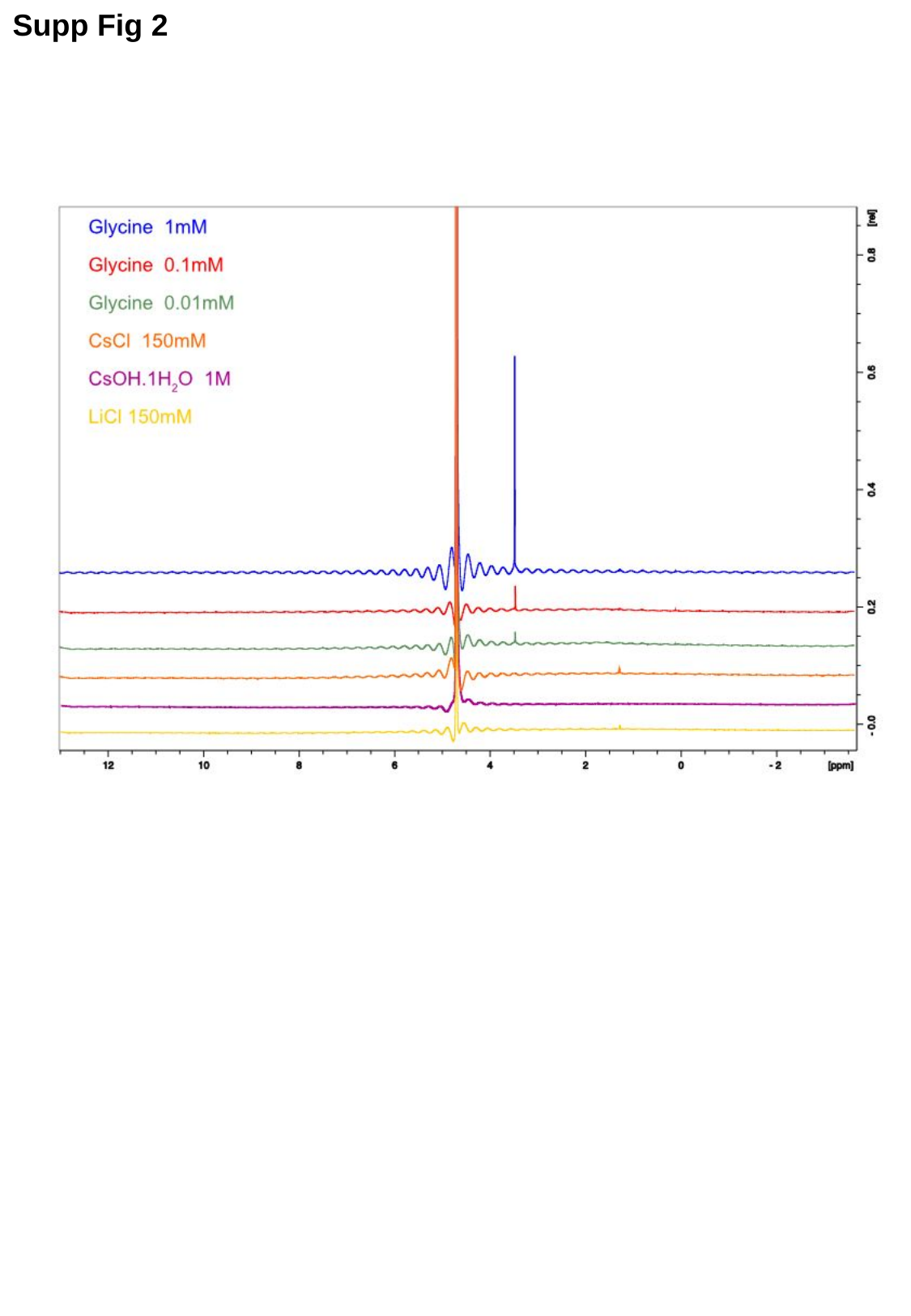

Supp Fig 2

## Slide 3
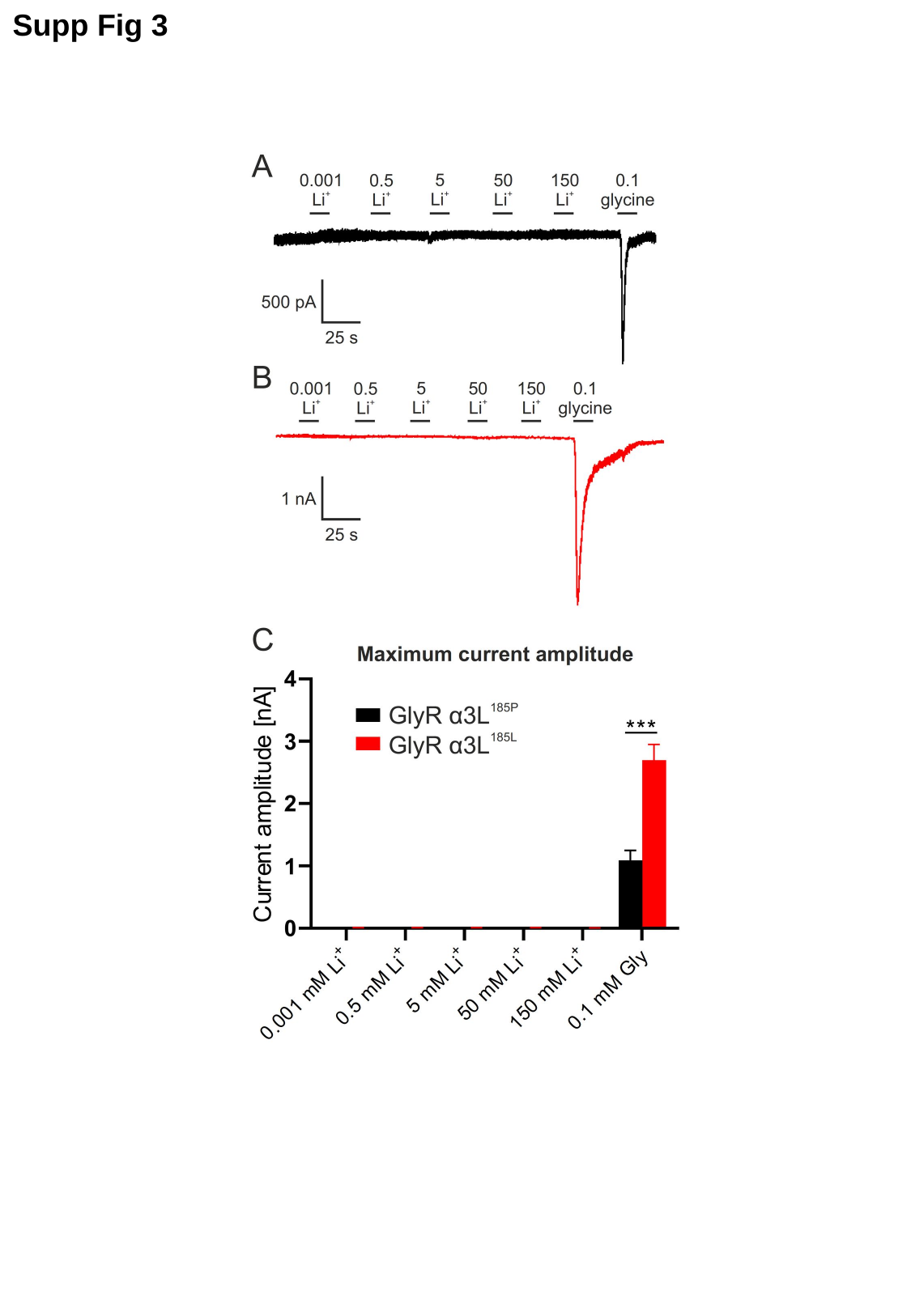

Supp Fig 3

## Slide 4
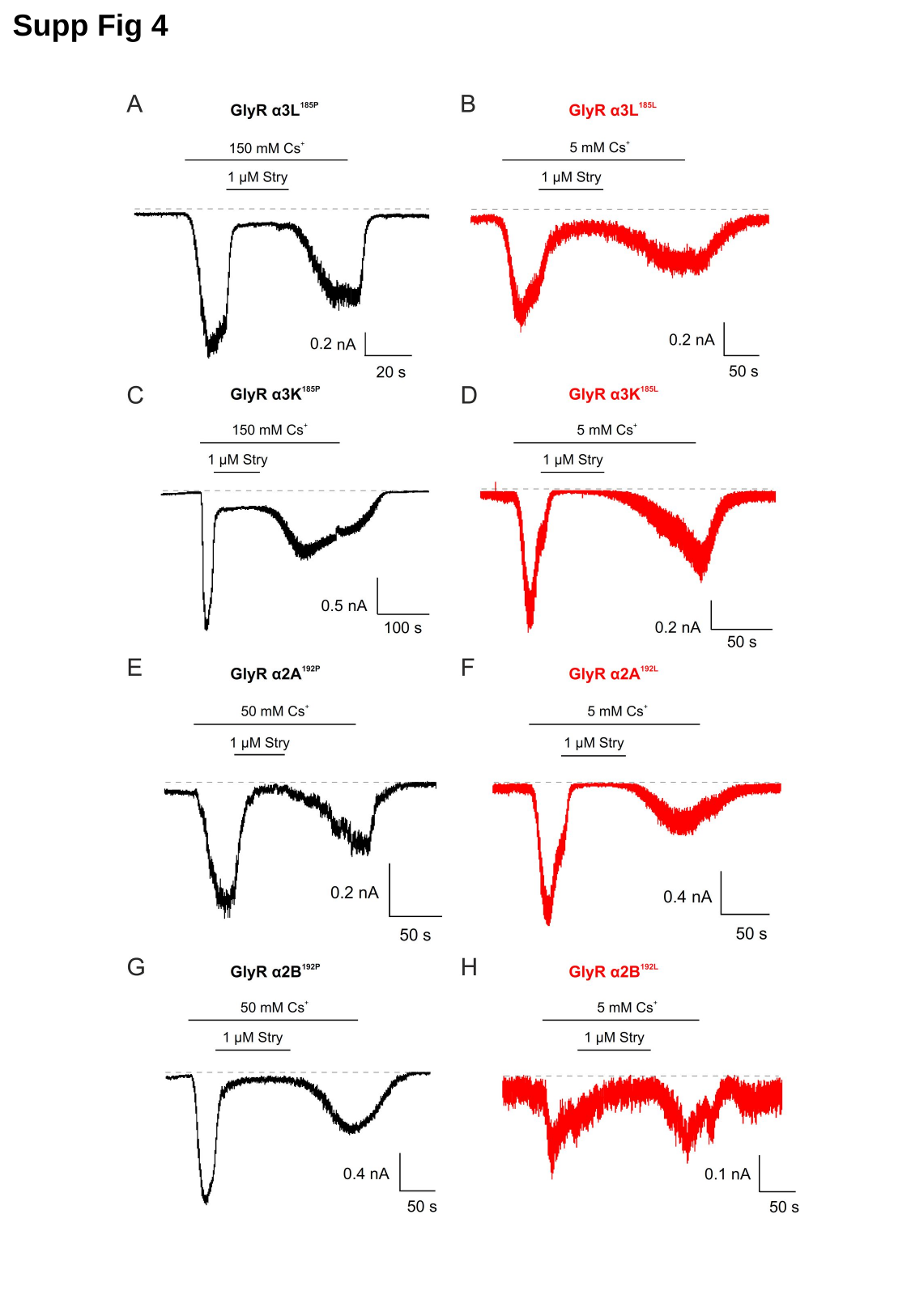

Supp Fig 4

## Slide 5
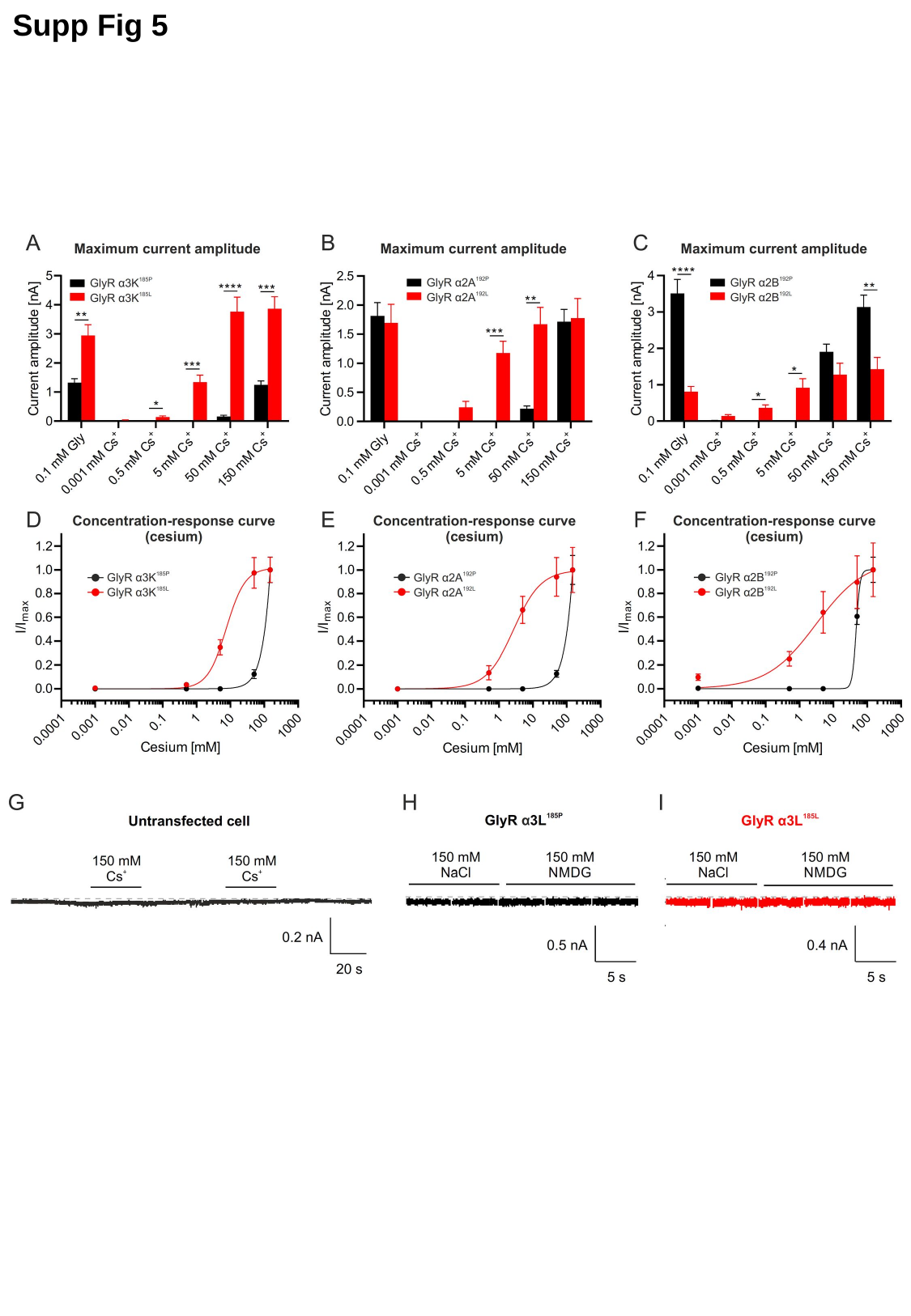

Supp Fig 5

## Slide 6
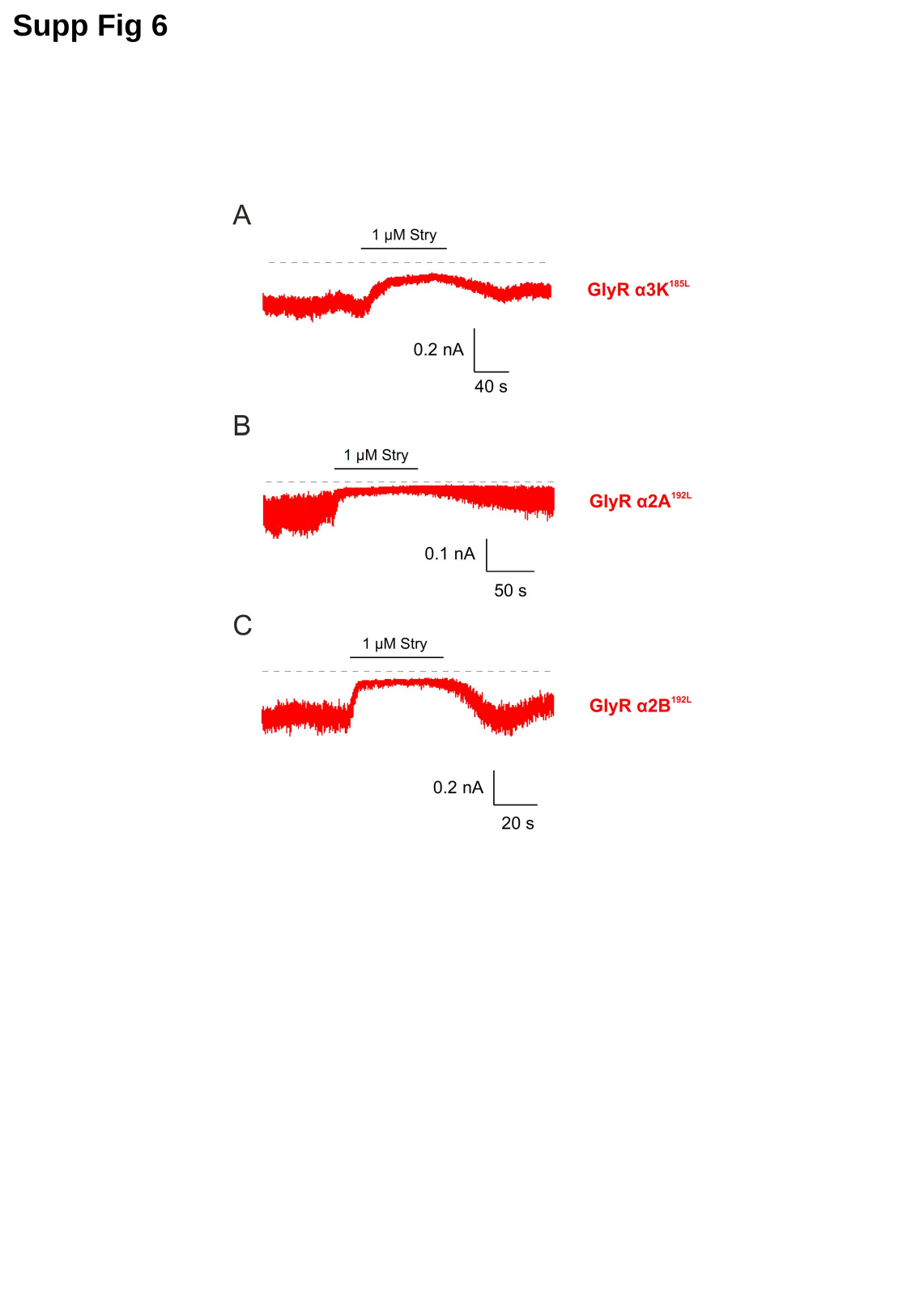

Supp Fig 6

## Slide 7
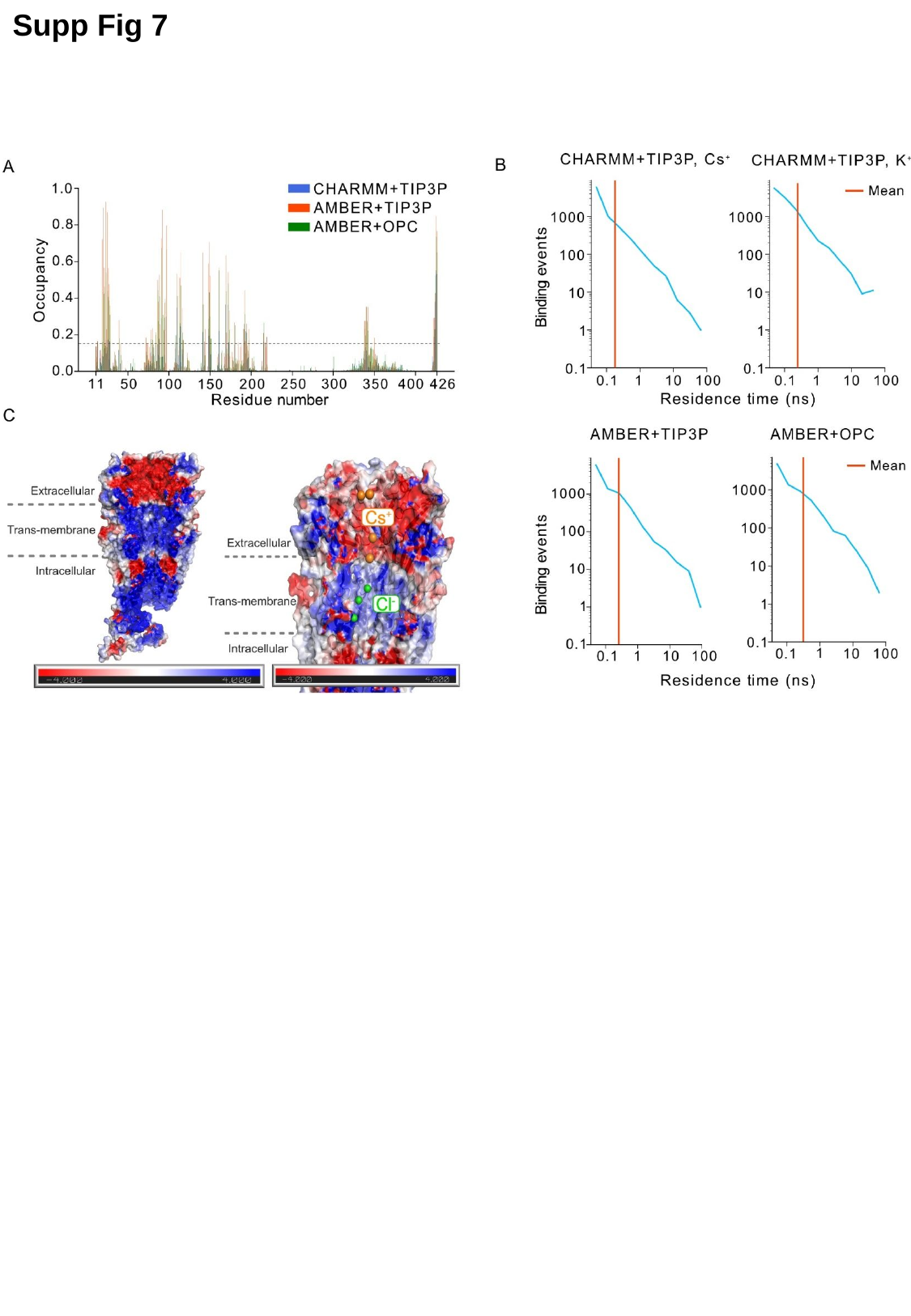

Supp Fig 7
